# Supplementary figures and images for: Drug repurposing for neurodegenerative diseases using Zebrafish behavioral profiles
Source: Biomed Pharmacother. Author manuscript; Available in PMC 2024 Mar 8. (PMC10922774; doi:10.1016/j.biopha.2023.116096)

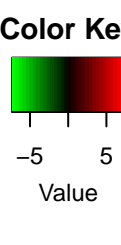

# Hierarchical Clustering

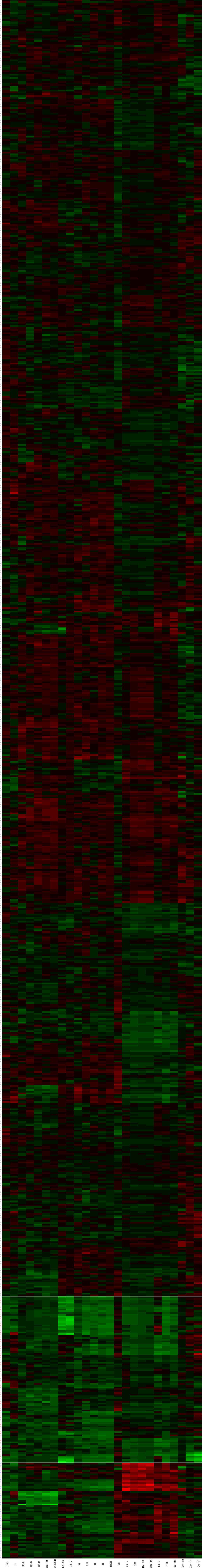

Supplement: Fig. S2 [file NIHMS1965884-supplement-Fig__S2.pdf]

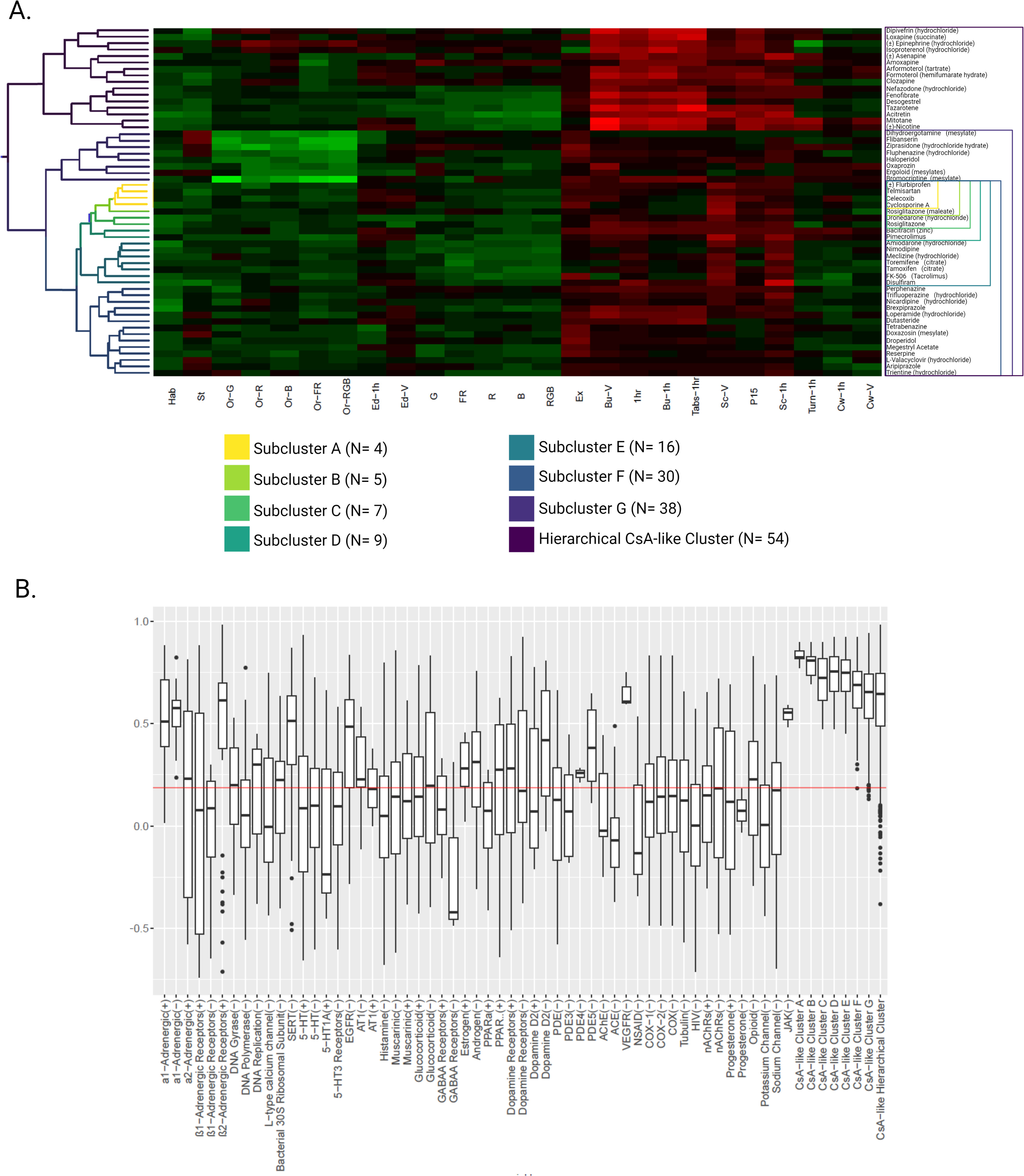

Supplement: Fig. S3 [file NIHMS1965884-supplement-Fig__S3.jpg]

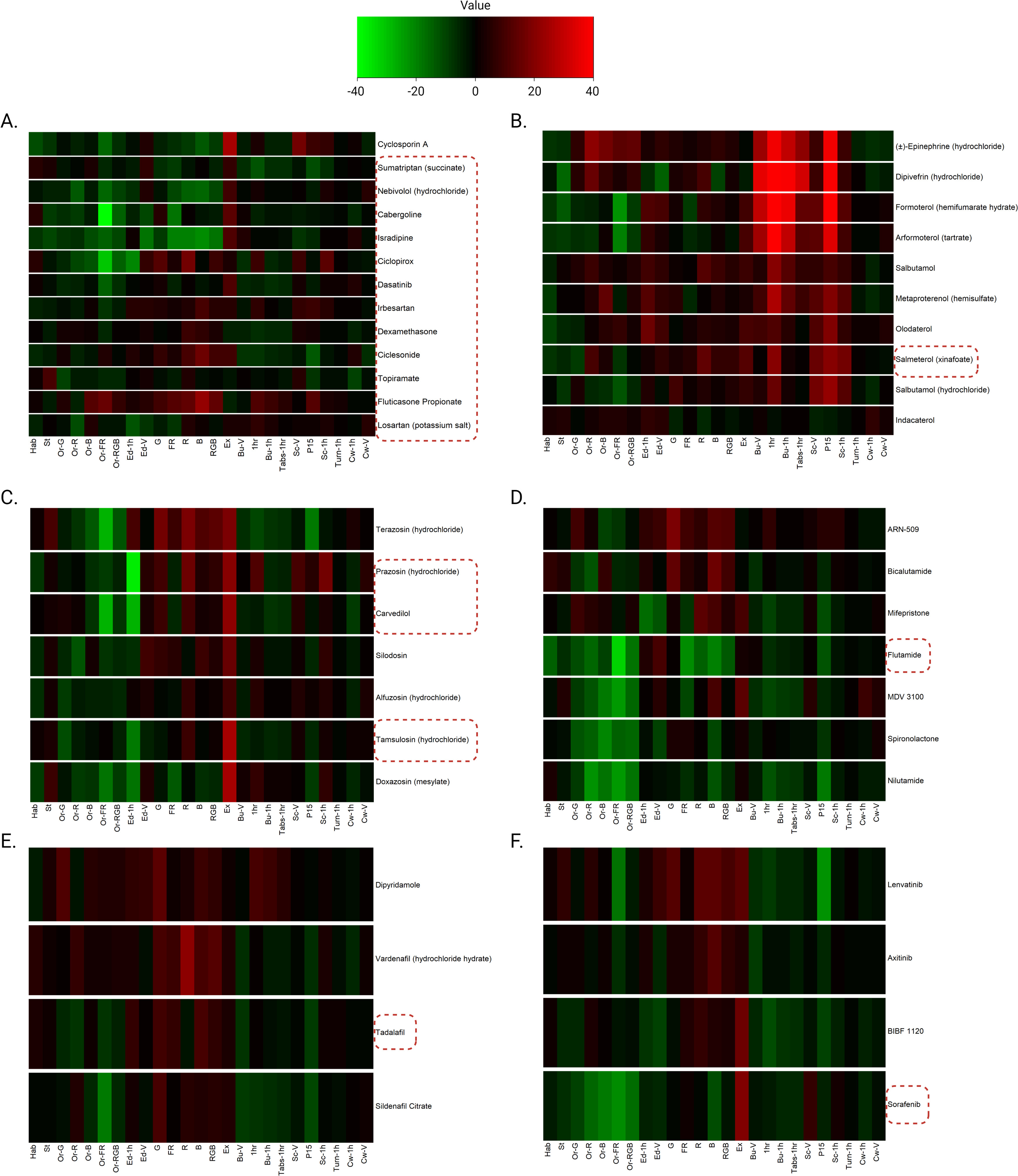

Supplement: Fig. S1 [file NIHMS1965884-supplement-Fig__S1.jpg]
